# Electronic cigarette use patterns and chronic health conditions among people experiencing homelessness in MN: a statewide survey

**DOI:** 10.1186/s12889-020-09919-4

**Published:** 2020-12-09

**Authors:** Eleanor L. S. Leavens, Becky R. Ford, Olamide Ojo-Fati, Tyler N. A. Winkelman, Katherine Diaz Vickery, Sandra J. Japuntich, Andrew M. Busch

**Affiliations:** 1 Department of Population Health, University of Kansas School of Medicine, 3901 Rainbow Blvd., Kansas City, KS 66160 USA; 2 Health, Homelessness, and Criminal Justice Lab, Hennepin Healthcare Research Institute, Minneapolis, MN USA; 3 Behavioral Health Equity Research Group, Hennepin Healthcare Research Institute, Minneapolis, MN USA; 4 General Internal Medicine, Department of Medicine, Hennepin Healthcare, Minneapolis, MN USA; 5 Department of Medicine, University of Minnesota, Minneapolis, MN USA; 6 Clinical Pharmacology, Department of Medicine, Hennepin Healthcare, Minneapolis, MN USA

## Abstract

**Background:**

Adults experiencing homelessness have higher rates of disease and premature morbidity compared to the general population. Tobacco use is a primary contributing factor to these disparities; however, less is known regarding e-cigarette use patterns among adults experiencing homelessness and whether e-cigarettes are used in a manner that is narrowing or widening health disparities. This study aimed to describe the 1) prevalence and trends in e-cigarette use, 2) correlates of e-cigarettes use, and 3) rates of chronic health conditions by product use pattern in a community-based sample of adults experiencing homelessness.

**Methods:**

Adults experiencing homelessness in Minnesota were surveyed by self-report in 2015 (*n* = 3672) and 2018 (*n* = 4181) regarding e-cigarette and combustible cigarette use, potential correlates of e-cigarette use, and self-reported chronic health conditions (i.e., asthma, hypertension, diabetes, and cancer).

**Results:**

Frequency of use increased from 2015 to 2018 for combustible cigarettes (66.9% vs. 72.3%), e-cigarettes (11.4% vs. 14.5%), and dual combustible/e-cigarette use (10.2% vs. 12.9%). The strongest bivariate correlates of past 30-day e-cigarette use were younger age, non-binary gender identification, non-heterosexual orientation, identification as White/Caucasian, greater frequency of lifetime homelessness, substance use, lack of regular place for medical care, mental health diagnosis, criminal justice involvement, and combustible cigarette smoking. Dual users had significantly higher rates of asthma and cancer than both those using combustible cigarettes and those using neither combustible nor e-cigarettes.

**Conclusions:**

During a time when cigarette smoking, e-cigarette use, and dual use were decreasing in the general population in Minnesota, rates increased in the homeless population. We observed that the rates of dual use were more than five times greater among homeless adults compared to the general population in 2018. Correlates of e-cigarette use were identified and should be used to identify subpopulations for intervention targeting. Mechanisms of the relationship between dual use and increased risks of health conditions deserve further study.

**Supplementary Information:**

The online version contains supplementary material available at 10.1186/s12889-020-09919-4.

## Background

Globally, it is estimated that over 150 million people experience homelessness [1]. In the United States (U.S.) alone, over half a million people experience homelessness on a given night [2]. Homelessness is associated with significant health disparities. All-cause mortality rates among people experiencing homelessness in the U.S. are 4.5 to 9.6-times higher than the general population [3] with higher incidence of drug overdose, substance use disorders, cancer, and heart disease driving disparities in mortality rates [3–5]. Tobacco use, particularly combustible cigarette smoking, also plays a significant role in widening health disparities between people experiencing homelessness and the general population. During a time when combustible tobacco use is at an all-time low in the U.S. general population, tobacco use remains common among people experiencing homelessness, with approximately three-quarters reporting current smoking [6]. However, less is known regarding electronic cigarette (e-cigarette) use among this population and the role it might play in widening or narrowing disparities. E-cigarette use is increasingly common among the general population in the U.S., with close to 7 million adult users [7]. Pod-based e-cigarettes, such as JUUL, entered the U.S. market in 2015 and have only accelerated the proliferation and popularity of e-cigarettes [7].

While current data suggest that e-cigarettes may be less harmful than combustible cigarettes [8], e-cigarettes are not harmless. Studies have shown e-cigarettes can deliver harmful and potentially harmful constituents including heavy metals, volatile organic compounds, and low levels of polycyclic aromatic hydrocarbons [9] and most contain highly-addictive nicotine [10, 11]. Dual use of combustible cigarettes and e-cigarette may be particularly harmful, especially to cardiovascular and respiratory health [9, 12, 13], with preliminary data showing potential associations between dual use and the pathophysiological respiratory changes associated with chronic obstructive pulmonary disease [12, 14] and cardiovascular disease [13]. If rates of dual use among the people experiencing homelessness are high, this could be contributing to widening health disparities among this vulnerable population. Conversely, if people experiencing homelessness are fully transitioning from combustible smoking to less harmful e-cigarette use, this pattern of use may reduce health disparities. Thus, continued monitoring and investigation is warranted.

Few studies have investigated e-cigarette use among adults experiencing homelessness [15–18]. The limited data that do exist indicate rates of current e-cigarette use among homeless smokers of 12 to 51% [6, 15–18] with the highest estimates from samples limited to youth and young adult smokers [16, 17]. However, all existing data are from relatively small (< 500) samples, were collected in 2016 or earlier (and thus may not capture the full influx of pod devices onto the market which now account for over 70% of the retail e-cigarette market) [19], and do not report on changes in e-cigarette use rates over time. Further, these studies included only current tobacco users, which is problematic because it does not allow for estimates of rates of e-cigarette use patterns among all people experiencing homelessness (e.g., there is no data on the rate of exclusive e-cigarette use in this population).

There is minimal existing data on correlates of e-cigarette use among those experiencing homelessness. A 2014 survey of adults experiencing homelessness who smoke in Boston [15] found that subsistence difficulties (i.e., problems finding shelter, food, clothing, a place to wash/go to the bathroom) and younger age were associated with e-cigarette use. Similarly, a 2017–2018 survey of youth/young adults experiencing homelessness aged 13–25 in Los Angeles county [17] found that being around others who use e-cigarettes, ease of accessibility compared to combustible cigarettes, identifying as lesbian, gay, bisexual, transgender, queer, or asexual (LGBTQA), and symptoms of depression were related to higher likelihood of e-cigarette use. A better understanding of e-cigarette use correlates would allow for more targeted prevention and treatment efforts.

The current study aims, in an exploratory fashion, to understand 1) trends of e-cigarette and dual e-cigarette and combustible cigarette use among adults experiencing homeless from 2015 to 2018, 2) the correlates of e-cigarette use among participants in 2018, and 3) frequency of chronic health conditions by use trajectory (i.e., non-smokers, combustible cigarette smokers, e-cigarette users, and dual users) in the 2018 sample. The study uses a large, state-wide survey of adults experiencing homelessness in Minnesota to explore these questions.

## Methods

### Data source and population

The Minnesota Homeless Study is a one-day, statewide person count and survey conducted every 3 years by the Amherst H. Wilder Foundation (St. Paul, MN). The purpose of this survey is to better understand the prevalence of homelessness in Minnesota and increase understanding of the life circumstances and health of this population. The Minnesota Homeless Study attempts to count all people staying in emergency and domestic violence shelters and transitional housing programs as well as people using outreach services (e.g. hot meal programs, service centers) but staying outside, doubled up with friends/family, and in encampments. Data from American Indian reservations are not included in our sample as they are managed separately.

A subset of willing people who are counted are verbally administered a survey by trained data collectors which takes approximately 1 h. These individuals receive a $10 gift card. We included adult survey respondents (18 years or older) from the latest two surveys, 2015 and 2018. All analyses are cross-sectional (i.e., we did could not link individuals who completed both surveys). The 2015 survey was conducted on October 22, 2015 and included 3672 adults. The 2018 survey was conducted on October 25, 2018 and included 4181 adults. More information on the count and survey methods is available at http://mnhomeless.org/. The authors had access to the data through a data use agreement with the Wilder Foundation. The Hennepin Healthcare Research Institute Institutional Review Board deemed this study exempt from review as it uses de-identified data. All analyses were conducted in STATA 15.1 (College Station, TX).

### Key variables

In both the 2015 and 2018 surveys, participants self-reported past 30-day use of “cigarettes” and of “e-cigarettes or a vaporizer” (“During the last 30 days have you used…” ; yes/no). We defined “dual use” as use of both e-cigarettes and combustible cigarettes in the past 30 days. All sociodemographic variables in Table 1 were collected by self-report.

**Table 1:** Bivariate comparisons by 30-day e-cigarette use status among 2018 sample – M (SD)/N(%)

Variable	E-cigarette use status		
	Past 30-day user (*n* = 607)	Past 30-day non-user (*n* = 3541)	*p*
Age	33.8 (12.4)	40.8 (13.6)	<.001
Gender			<.001
Male	345 (56.8)	1920 (54.2)	
Female	252 (41.5)	1606 (45.4)	
Other	10 (1.7)	14 (0.4)	
Transgender identity			.022
Yes	12 (2.0)	33 (0.9)	
No	588 (98.0)	3467 (99.1)	
Sexual orientation			<.001
Straight/heterosexual	507 (84.6)	3188 (91.5)	
Non- heterosexual^1^	92 (15.4)	298 (8.5)	
Race			<.001
White or Caucasian	268 (45.0)	1277 (36.6)	
African American/African	151 (25.3)	1272 (36.5)	
American Indian/Alaska Native	79 (13.3)	535 (15.3)	
Asian or Pacific Islander	9 (1.5)	61 (1.8)	
Other	89 (14.9)	343 (9.8)	
Ethnicity			.032
Hispanic	62 (10.4)	271 (7.8)	
Non-Hispanic	537 (89.6)	3224 (92.2)	
Education			.138
No high school diploma	253 (41.7)	1360 (38.5)	
High school diploma/GED	354 (58.3)	2172 (61.5)	
Employment			.026
Employed	185 (30.5)	920 (26.2)	
Not employed	422 (69.5)	2598 (73.8)	
Duration of homelessness (lifetime)			.901
Less than 1 year	244 (41.0)	1416 (40.5)	
1–5 years	248 (41.7)	1478 (42.3)	
More than 5 years	103 (17.3)	599 (17.2)	
Frequency of homelessness (lifetime)			<.001
1 time	100 (16.9)	818 (23.8)	
2–4 times	187 (31.6)	1225 (35.6)	
5–7 times	119 (20.1)	525 (15.2)	
8 or more times	185 (31.3)	875 (25.4)	
Substance use (past 30-day)			
Alcohol			<.001
Yes	283 (46.9)	1207 (34.2)	
No	320 (53.1)	2326 (65.8)	
Marijuana			<.001
Yes	294 (48.8)	978 (27.7)	
No	308 (51.2)	2554 (72.3)	
Other illicit substance use			<.001
Yes	194 (32.3)	652 (18.5)	
No	407 (67.7)	2879 (81.5)	
Health Insurance			.288
Yes	459 (77.8)	2656 (75.8)	
No	131 (22.2)	849 (24.2)	
Have regular place for medical care			<.001
Yes	380 (63.1)	2479 (70.3)	
No	222 (36.9)	1049 (29.7)	
Mental health diagnosis from provider (past 24 months)			<.001
Yes	479 (79.7)	2309 (65.6)	
No	122 (20.3)	1210 (34.4)	
Criminal Justice Involvement (past 12 months)			<.001
Yes	201 (33.1)	814 (23.0)	
No	406 (66.9)	2727 (77.0)	
Cigarette smoking (past 30-day)			<.001
Yes	539 (88.8)	2482 (70.1)	
No	68 (11.2)	1058 (29.9)	

*Note.* Percentages exclude missingness on that item. ^1^reported gay, lesbian, bisexual, or wrote in another identification

### Statistical analysis

We examined rates of combustible cigarette smoking, e-cigarette use, and dual use in 2015 and 2018 and present these trends alongside previously published statewide data from the general population [20, 21]. We also assessed bivariate correlates of e-cigarette use in the 2018 data. Bivariate comparisons were made using independent samples t-tests (for continuous data), Chi Square tests (for categorical data), and Mann-Whitney U tests (for ordinal data). In this analysis, we compared e-cigarette users (past 30-day) to non-users to describe the overall characteristics of past 30-day users.

Finally, we compared frequency of self-reported chronic health conditions in the past-year among non-smokers, combustible cigarette smokers, e-cigarette users, and dual users in the 2018 sample. Participants self-reported past-year rates of the following chronic health conditions: 1) Asthma 2) Hypertension 3) Cancer 4) Diabetes (“During the last 12 months, did you have any of the following illnesses, conditions, or problems?”; yes/no). Estimates controlled for age and gender using multivariable logistic regression. Adjusted probabilities were obtained using predictive margins with covariates held at observed sample values. We compared these four tobacco use patterns to contrast dual use with other groups and to allow for comparisons to the existing literature in the general population.

## Results

**Fig. 1:**
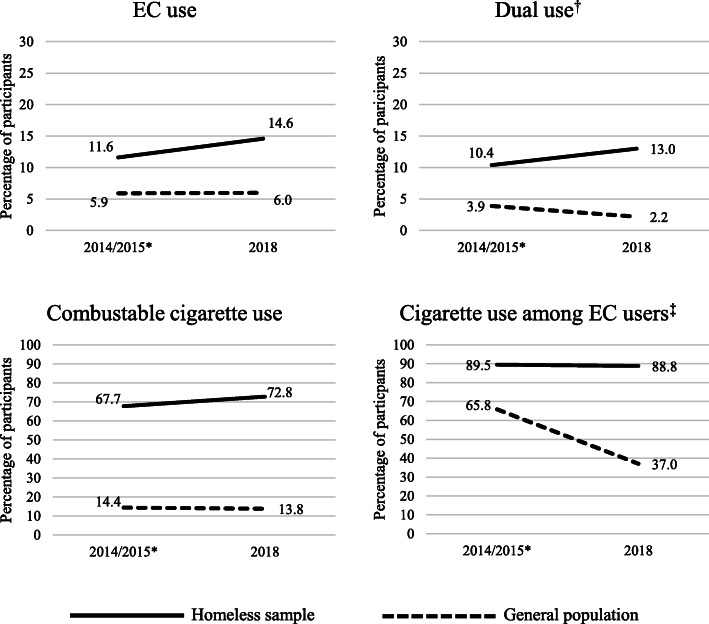
Rates of e-cigarette use, combustible cigarette smoking, dual use, and combustible cigarette smoking among e-cigarette users in homeless adults and the general population in Minnesota during 2014/2015 and 2018. *Note.* * Homeless sample data were collected in 2015, while the general population data were collected in 2014. General population data have been previously reported by Clearway Minnesota, while the homeless sample data are reported as part of the current investigation. EC = electronic cigarette. ^†^Refers to the percentage of participants who report both e-cigarette use and cigarette smoking in the past month. ^‡^Refers to the percentage of past month e-cigarette users that report past month cigarette smoking

In the 2015 survey, 3627 adults (98.8% of respondents) completed the e-cigarette use question, while 4148 (99.2% of respondents) did so in the 2018 survey. Four hundred twenty-one participants (11.6%) used e-cigarettes in the 2015 survey, while 607 (14.6%) used in the 2018 survey. Two thousand four hundred fifty-six participants (67.7%) used combustible cigarettes in the 2015 survey, while 3021 (72.8%) used in the 2018 survey. Three hundred seventy-seven participants (10.4% of all participants) were dual users in the 2015 survey, while 539 (13.0% of all participants) reported dual use in the 2018 survey. Thus, among those that used e-cigarettes, the rate of combustible cigarette smoking was 89.5% (377 of 421) in 2015 and 88.8% (539 of 607) in 2018. These data are contrasted with data from the general population in Minnesota gathered in 2014 and 2018 in Fig. 1.

Table 1 presents bivariate correlates of e-cigarette use in 2018. Missing data for all variables in Table 1 ranged from 0.0–2.7%. Percentages and statistical testing reported exclude missing data on that variable. Bivariate comparisons indicated that sociodemographic variables including younger age, gender (driven by higher rates among those non-binary gender identifying), identifying as transgendered, non-heterosexual orientation (i.e., reported gay, lesbian, bisexual, unsure, or wrote in another identification), race (driven by higher rates among Whites/Caucasians), Hispanic ethnicity, employment, and greater frequency of lifetime homelessness were related to past 30-day e-cigarette use at *p* < .05. Past 30-day use of all substances (i.e., alcohol, marijuana, other illicit drug use, and combustible cigarette smoking) were significantly associated with past 30-day e-cigarette use (*p* < .05). Finally, lack of regular place for medical care, mental health diagnosis in the past 2 years, and involvement in the criminal justice system over the last 12 months were related to current e-cigarette use at *p* < .05.

In analyses assessing the association between product use status (non-use, e-cigarette use, combustible cigarette smoking, and dual use) and past year chronic health conditions, dual users had the highest rates of asthma and cancer compared to all other groups. Specifically, all use groups had higher rates of asthma compared to non-users (all *p* < .05) and dual users showed higher rates compared to combustible cigarette smokers (*p* = .01). Combustible cigarette smokers and dual users showed higher rates of cancer compared to non-users (all *p* < .05) and dual users had higher rates compared to combustible cigarette smokers (*p* = .028). Combustible cigarette smokers showed lower rates of diabetes compared to non-users (*p* = .021). No other groups differed in their rates of diabetes. No groups differed in their rates of hypertension. See Table 2 and the supplementary table for complete results.

**Table 2:** Rates of self-reported chronic health conditions among those using use categories

	% [95% CI]			
	None	Combustible only	E-cigarette only	Dual use
Asthma	14.7 [12.7,16.8]	21.0 [19.4,22.6]^a^	25.4 [15.1, 35.8]^a^	28.0 [24.2, 31.8]^ab^
Hypertension	28.6 [25.9,31.2]	27.0 [25.4,28.78]	21.9 [10.7, 33.0]	28.9 [24.9, 32.9]
Diabetes	13.6 [11.6,15.7]	10.9 [9.7,12.0]^c^	13.4 [3.8,22.9]	11.5 [8.4,14.5]
Cancer	1.6 [0.8,2.3]	2.7 [2.0,3.3]^a^	2.4 [−2.2,7.1]	5.2 [3.0,7.5]^ab^

Note: ^a^ Significantly higher rate than none. ^b^ Significantly higher rate than combustible only. ^c^ Significantly lower rate than none

## Discussion

Previously published statewide general population data indicate that the past month e-cigarette use rate among adults in Minnesota was 5.9% in 2014 and remained steady at 6.0% in 2018 [20, 21]. In contrast, we found that rates of e-cigarette use among adults experiencing homelessness in Minnesota are both significantly higher and appear to be increasing over a similar timeframe. While the reasons for this increase in e-cigarette use among adults experiencing homelessness remain unknown, there appear to be differences in reasons for use between the two populations. Studies suggest that homeless adults use e-cigarettes to avoid having to go outside and to deal with situations where they cannot smoke [16] while the general population uses e-cigarettes for cessation or health-related reasons [22].

Past month dual e-cigarette and combustible cigarette use in the general Minnesota population was 3.9% in 2014 and dropped to 2.2% in 2018 [20, 21]. In contrast, we found that dual use rates among adults experiencing homelessness in Minnesota were higher and increased significantly over a similar timeframe. Two comparisons from 2018 are particularly striking. First, the dual use rate in the homeless sample was 13.0% in 2018, which is more than five times higher than the general population rate in the same year. Second, in the homeless sample, *majority* (88.8%) of e-cigarette users were also smoking combustible cigarettes, while a *minority* (37.0%) of e-cigarette users in the general population sample were also smoking combustible cigarettes. The most recent laboratory and epidemiological data suggest that while switching completely from combustible cigarettes to e-cigarettes could confer significant harm reduction, using both products may be significantly more harmful than using combustible cigarettes alone due to increased toxicant exposure [9, 12, 13], particularly if dual use does not result in reduced cigarette smoking [9]. Thus, the general population in Minnesota may be experiencing some harm reduction from e-cigarette use, while use of the same product may be *increasing* harm in the homeless population. If this is the case, dual use of e-cigarettes and combustible cigarettes may have a role in increasing existing tobacco related health disparities experienced by adults experiencing homelessness. Our findings are consistent with recent work suggesting that smokers with lower socioeconomic status are less likely to use e-cigarettes in a manner associated with harm reduction or cessation [23].

The significant bivariate correlates of e-cigarette use indicate the subpopulations that should be of particular interest for public health and intervention targeting. It is notable that each gender/sexual minority category, each substance abuse category, having a mental illness, and recent criminal justice involvement were all associated with a > 50% increase in e-cigarette use rate. Our findings are in line with previous data indicating young age and mental illness as strong predictors of e-cigarette use among the homeless [6, 15, 17]. Most of the correlates identified in the current study have been previously identified as correlates in general population samples [24, 25], suggesting consistency in sociodemographic and social factors associated with e-cigarette use among homeless and general populations. Given these similarities, reasons for use and effective interventions for e-cigarette use may be similar as well. To our knowledge this is the first study to report criminal justice involvement as a predictor of e-cigarettes use. This is important as those with criminal justice involvement have significant heart and lung disease disparities relative to the general population [26–28] due at least in part to high rates of combustible tobacco use [28].

Analyses of the association between use status and past year physical chronic health conditions showed that dual users had higher rates of cancer and asthma compared to the other groups. These data add to a growing body of literature suggesting that dual use of e-cigarettes and combustible cigarettes is associated with higher concurrent rates of health problems than exclusive cigarette use, exclusive e-cigarette use, and no use [9, 12, 13, 29]. The mechanisms of this association remain largely unknown. It is possible that adding e-cigarettes to combustible cigarette smoking is causing increased rates of chronic health conditions and one study has suggested that dual use increases risk for developing a respiratory disease (i.e., chronic obstructive pulmonary disease, chronic bronchitis, emphysema, or asthma) at 1–2 year follow-up [14]. However, it is also possible that the chronic health condition diagnosis may predate the dual use pattern. It may be the case that smokers with a chronic health condition are more likely to engage in e-cigarette use as an attempt to reduce harm related to that condition. There is some evidence for this temporal pattern among cardiac patients with data showing cardiac patients who smoked viewed e-cigarettes as less harmful than smoking and a significant proportion initiated e-cigarette use following a cardiac event [30]. Future studies should examine these associations prospectively over longer timelines.

The results of the current study should be considered with limitations in mind. The sample was drawn from a single state in the U.S. with a relatively low smoking prevalence [31] and therefore cannot be directly generalized to the broader adult homeless population. Use status was self-reported and the data available on e-cigarette and other tobacco use was limited (e.g., type of e-cigarette device was not assessed; non-combustible tobacco use was not assessed). Additionally, the data are cross-sectional; therefore, temporal associations, particularly in terms of tobacco use and onset of chronic health conditions, are not possible. The data are self-reported amongst a population with, on average, low health literacy which may have reduced reliability. However, this limitation is mitigated by the interview methods employed in survey administration and data suggesting that people experiencing homelessness are able to report on their health conditions with a high degree of accuracy [32]. Finally, it was not possible to determine if dual users were using e-cigarettes to reduce combustible tobacco use or if those only using e-cigarettes had switched from combustible tobacco use, which would allow for a more fine-grained risk vs. benefit analysis of the effect of e-cigarettes on this population.

## Conclusions

The current study is among the first to describe e-cigarette, combustible cigarette, and dual use patterns among people experiencing homelessness. Findings suggest that, during a time when cigarette smoking, e-cigarette use, and dual use were decreasing in the general population in Minnesota, rates increased in the homeless population. In addition, rates of dual use were almost five times higher in the homeless in 2018 compared to the general population and was associated with increased rates of past year chronic health conditions (i.e., asthma and cancer). E-cigarettes, driven by high rates of dual use, may be contributing to the already significant health inequalities among people experiencing homelessness. Future research on e-cigarette use among individuals experiencing homeless is warranted.

## Supplementary Information

**Additional file 1: Table**. Differences in rates of self-reported chronic health conditions between use categories

## Data Availability

The datasets analyzed during the current study are not publicly available. The authors had access to the data through a data use agreement with the Wilder Foundation. Any reasonable request for the data underlying these analyses will be considered in consultation with the Wilder Foundation.

## Abbreviations

U.S: United States
LGBTQA: Lesbian, gay, bisexual, transgender, queer, or asexual
MN: Minnesota
