# Supplementary material for: Electronic cigarette use patterns and chronic health conditions among people experiencing homelessness in MN: a statewide survey
Source: BMC Public Health. 2020 Dec 9;20:1889. doi: 10.1186/s12889-020-09919-4 (PMC7724886; doi:10.1186/s12889-020-09919-4)
Supplement: Supplementary file 1 — Additional file 1: Table. Differences in rates of self-reported chronic health conditions between use categories [file 12889_2020_9919_MOESM1_ESM.docx]

Supplementary material

Table: Differences in rates of self-reported chronic health conditions between use categories

|  | % [95% CI] | | | |  |
| --- | --- | --- | --- | --- | --- |
|  | None vs. Combustible | None vs. E-cigarette | None vs. Dual | Combustible vs. E-cigarette | Combustible vs. Dual |
| Asthma | -.06 [-.09,-.04]^c^ | -.11 [-.21,.00]^a^ | -.13 [-.18,-.09]^c^ | -.04 [-.14,.06] | -.07[-.11,-.03]^b^ |
| Hypertension | .01 [-.02,.05] | .09 [-.03,.20] | .00 [-.05,.04] | .07 [-.04,.18] | -.02 [-.06,.02] |
| Diabetes | .03 [.00,.05]^a^ | .00 [-.10,.10] | .02 [-.02,.06] | -.03 [-.13,.07] | -.01 [-.04,.02] |
| Cancer | -.01 [.02,.00]^a^ | -.01 [-.06,.04] | -.04 [-.06,-.01]^b^ | .00,[-.05,.05] | -.02[-.05,.00]^a^ |

Note: ^a^ *p* <.05 ^b^ *p* <.01 ^c^ *p* <.001
